# Browsing Multiple Subjects When the Atlas Adaptation Cannot Be Achieved *via* a Warping Strategy

**DOI:** 10.3389/fninf.2022.803934

**Published:** 2022-03-03

**Authors:** Denis Rivière, Yann Leprince, Nicole Labra, Nabil Vindas, Ophélie Foubet, Bastien Cagna, Kep Kee Loh, William Hopkins, Antoine Balzeau, Martial Mancip, Jessica Lebenberg, Yann Cointepas, Olivier Coulon, Jean-François Mangin

**Affiliations:** ^1^Université Paris-Saclay, CEA, CNRS UMR 9027, Baobab, NeuroSpin, Gif-sur-Yvette, France; ^2^PaleoFED Team, UMR 7194, CNRS, Département Homme et Environnement, Muséum National d’Histoire Naturelle, Musée de l’Homme, Paris, France; ^3^INT - Institut de Neurosciences de la Timone, Aix-Marseille Univ, CNRS UMR 7289, Marseille, France; ^4^Department of Comparative Medicine, University of Texas MD Anderson Cancer Center, Bastrop, TX, United States; ^5^Department of African Zoology, Royal Museum for Central Africa, Tervuren, Belgium; ^6^Maison de la Simulation, CNRS, CEA Saclay, Gif-sur-Yvette, France; ^7^Université de Paris, INSERM UMR 1141, NeuroDiderot, Paris, France

**Keywords:** visualization, brain atlas, inter-subject, 3D, parcellation atlas, structural approach

## Abstract

Brain mapping studies often need to identify brain structures or functional circuits into a set of individual brains. To this end, multiple atlases have been published to represent such structures based on different modalities, subject sets, and techniques. The mainstream approach to exploit these atlases consists in spatially deforming each individual data onto a given atlas using dense deformation fields, which supposes the existence of a continuous mapping between atlases and individuals. However, this continuity is not always verified, and this “iconic” approach has limits. We present in this study an alternative, complementary, “structural” approach, which consists in extracting structures from the individual data, and comparing them without deformation. A “structural atlas” is thus a collection of annotated individual data with a common structure nomenclature. It may be used to characterize structure shape variability across individuals or species, or to train machine learning systems. This study exhibits *Anatomist*, a powerful structural 3D visualization software dedicated to building, exploring, and editing structural atlases involving a large number of subjects. It has been developed primarily to decipher the cortical folding variability; cortical sulci vary enormously in both size and shape, and some may be missing or have various topologies, which makes iconic approaches inefficient to study them. We, therefore, had to build structural atlases for cortical sulci, and use them to train sulci identification algorithms. *Anatomist* can display multiple subject data in multiple views, supports all kinds of neuroimaging data, including compound structural object graphs, handles arbitrary coordinate transformation chains between data, and has multiple display features. It is designed as a programming library in both C++ and Python languages, and may be extended or used to build dedicated custom applications. Its generic design makes all the display and structural aspects used to explore the variability of the cortical folding pattern work in other applications, for instance, to browse axonal fiber bundles, deep nuclei, functional activations, or other kinds of cortical parcellations. Multimodal, multi-individual, or inter-species display is supported, and adaptations to large scale screen walls have been developed. These very original features make it a unique viewer for structural atlas browsing.

## Introduction

### Brain Atlases in Brain Research and Neurosciences

The idea of “functional specialization” or “**segregation**” is central to our current understanding of brain organization (Tononi et al., 1994). It is usual to consider that the brain of each species can be subdivided into elementary macroscopic entities transposable from one individual to another (Eickhoff et al., 2015, 2018; Arslan et al., 2018; Sotiropoulos and Zalesky, 2019; Moghimi et al., 2021). The number of such entities to be matched across subjects, however, is large and undetermined, especially for the human cortex, which creates many practical difficulties. For the human cortex, these difficulties result from our limited understanding of its subdivisions but also from the variability of its morphology. Despite a relatively stable folding topography from one individual to another, the shape of the sulci varies enormously, which hinders their use as landmarks to identify the exact localization of elementary architectural features (Mangin et al., 2016). Furthermore, the exact relationship between these complex shapes and the topography of cortical areas is only partially understood today (Amiez et al., 2006; Sun et al., 2016; Bodin et al., 2018; Mangin et al., 2019; Eichert et al., 2021).

For group studies, the mainstream strategy of the brain imaging community consists in **building atlases** of the brain organization that can be adapted to any brain using “spatial normalization” (Lancaster et al., 2010; Evans et al., 2012; Amunts et al., 2014). Brain atlases, however, can be of very varied natures. A brain atlas can be an image of the brain or a map where the positions, extents, shapes, and relative topography of the structures of interest are represented. Brain atlases can be built from one single subject [Colin27 (Holmes et al., 1998), AAL (Tzourio-Mazoyer et al., 2002), BigBrain (Amunts et al., 2013)], or from a set of subjects [ICBM152 (Fonov et al., 2009), Julich Brain (Amunts et al., 2020)]. Hence, brain atlases can include information about inter-subject variability (average maps, probabilistic maps, variance maps, etc.) (Evans et al., 2012; Amunts et al., 2014, 2020). Data from different modalities are generally represented using different atlases. They can come from different scales of observation, from microscopic to macroscopic. Some are volume based (Holmes et al., 1998; Tzourio-Mazoyer et al., 2002; Fonov et al., 2009; Amunts et al., 2013, 2020; Fan et al., 2016); others are surface based, like cortical atlases (Yeo et al., 2011; Auzias et al., 2016; Glasser et al., 2016; Lefranc et al., 2016), or made of fiber pseudo-trajectories (Guevara et al., 2012, 2017). The Human Brain Project, a flagship of the European Union, is designing an integrated multilevel brain atlas bridging the standard reference spaces used by the community (Lebenberg et al., 2018; Amunts et al., 2019).

**FIGURE 1:**
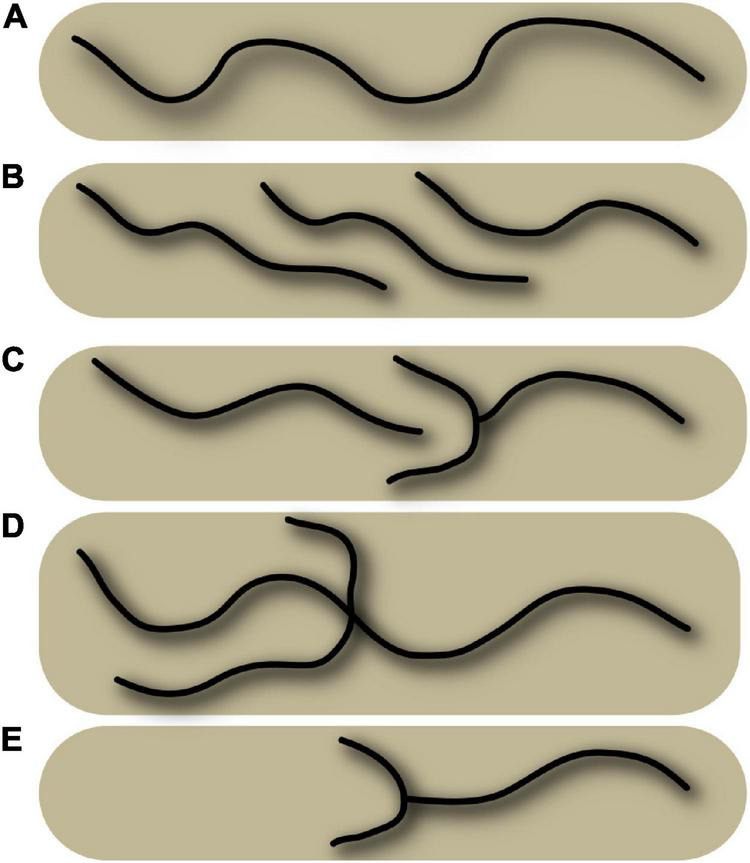
Schematic representation of various configurations of a sulcus, which illustrates the absence of general point-to-point matching between subjects. The schema represents five superior frontal sulci, and some configurations can be observed in real data in Figure 5. **(A)** Single, long sulcus configuration. **(B)** Interrupted sulcus with parallel overlaps. **(C)** Interrupted sulcus with small branches. **(D)** Sulcus with a short branch and a long branch, located at a different position from configuration **(C)**. **(E)** Shorter sulcus with small branches and a missing part. Superimposing the various situations here, even using non-linear warping, will not provide a perfect match.

To build a population-based atlas, or to align the atlases with each other or with new subjects, the mainstream strategy is to establish **point-to-point matching** between subjects or atlases, using dense, continuous coordinate transformations fields. This has led to develop a large number of registration and warping algorithms, which have taken a central role in neuroimaging research [DARTEL (Ashburner, 2007), ANTS (Avants et al., 2009), MSM (Robinson et al., 2014), and DISCO (Lebenberg et al., 2018), etc.]. This strategy, usually called **spatial normalization**, is based on a simple but approximate tenet according to which all the brains of a given species can be spatially transformed toward a reference space where their architectures are aligned. This paradigm has greatly contributed to the success of brain mapping because it allows the field to compare the results of all experiments. This paradigm, however, has limitations that may pose problems for some research programs. Spatial normalization does not really provide an architecturally compliant point-to-point correspondence between subjects (see Figure 1), which has some consequences (Klein et al., 2010; Glasser et al., 2016; Mangin et al., 2016). Some studies also show that brain networks have individual specificities (Domhof et al., 2021). Multi-subject atlases may overcome some difficulties for some applications, but even segmentation of morphological structures using non-linear alignment and multi-subject atlases seems less efficient than patch-based (Manjón and Coupé, 2016) or deep learning (Fang et al., 2019) strategies which do not use warping. In this study, we want to illustrate that the mainstream brain mapping strategy, sometimes called the “**iconic strategy,**” can be coupled with a so-called “**structural strategy,**” which does not adapt the atlases with a warping, but simply with annotations of domain-specific brain features from a nomenclature (Mangin et al., 2004b). In some ways, this is the usual strategy for conducting comparative studies across species, because it is often difficult to align brains of different species with an iconic approach.

Historically, we have been interested in this **structural strategy out of necessity**. Our research program aims to decipher the **variability of cortical folding patterns**. The topological variability of these patterns seemed inaccessible to us with an iconic approach, which led us to design a structural software world dedicated to this topic. Gradually, we realized that the structural strategy could be useful for other application areas, such as the mapping of fiber bundles, functional, or architectonic areas. When studying fine details and variability of a brain subdivision, it is generally not possible to establish a complete, reasonable, point-to-point correspondence between subjects because the studied structures vary too much or even have different topologies. Moreover, it is not desirable to apply strong warping that may often be arbitrary when the goal is to model the inter-subject variability.

In this study, we, therefore, advocate a different kind of atlas, which does not reduce the inter-subject variability to averages or probabilistic maps. Structural atlases embed the representations of a set of subjects in the spirit of **multi-subject atlases** (Aljabar et al., 2009; Lötjönen et al., 2010; Iglesias and Sabuncu, 2015) or the training sets of deep learning (Coupé et al., 2020; Henschel et al., 2020). The essence of the structural strategy consists in extracting first, during a preprocessing of the subject’s images, modality-specific objects [elementary brain structures like cortical folds (Mangin et al., 1995), cortical pits (Cachia et al., 2003; Im et al., 2010; Auzias et al., 2015), fascicles of pseudo-fibers with similar trajectories (Guevara et al., 2011), and regions homogeneous in terms of function (Coulon et al., 2000; Operto et al., 2012), etc.]. Then, these objects can be matched across subjects using a common nomenclature. In other words, the atlases we are using are **sets of individual high-level structural representations, annotated with a common nomenclature**. The usual multi-subject atlases are extreme cases where the annotation is performed at the lowest scale, corresponding to voxels. The structural strategy can be very useful during the construction of multi-subject atlases, especially when the common nomenclature has to be discovered. The manual annotation of high-level representations is more efficient than drawing, especially during an exploration stage, including trials and errors. Furthermore, this strategy can leverage the information embedded in the voxel-clustering operations performed during the preprocessing. These high-level representations can be used to design the automatic inference of the common nomenclature (Coulon et al., 2000; Im et al., 2010; Guevara et al., 2012, 2017; Operto et al., 2012). They can also be used to regularize the computer vision problem that consists in recognizing the structures corresponding to the nomenclature in new subjects (Riviere et al., 2002; Perrot et al., 2011; Borne et al., 2020). Although this strategy seems old-fashioned, as deep networks are now supposed to discover the best representations of the data, it can be efficient to fight the under-specification of end-to-end deep learning models (Borne et al., 2020; D’Amour et al., 2020).

In the following, we describe the functionalities of ***Anatomist***, the pillar of our software world dedicated to the visualization tasks specific to the structural approach to brain mapping. While most kinds of neuroimaging visualization software exploit the possibility to bring all the subjects to the same space, *Anatomist* lets each individual data live in its own native space, preserving it from warping operations modifying its shape. Inter-subject correspondences can be achieved *via* coordinate transformations, if needed, but mainly by structure correspondence. In our opinion, this strategy is mandatory for the questions related to structural variability, which cover topological variations in the shape of a specific entity or in the relative topography of a set of entities. *Anatomist* allows us to browse our structural atlases once they have been designed, but more importantly, *Anatomist* is often used to create such atlases *via* the labeling of individual structural representations.

While our annotated atlases used to train Machine Learning tools to recognize some brain entities in new subjects are currently limited to a few hundred subjects, they will probably grow in the future to provide a better representation of the general population. For this purpose, *Anatomist* can now be interfaced with wall-size screens, allowing seamless navigation through a large number of subjects according to the needs. Each visualized subject is endowed with a dedicated window. The navigation tools of Anatomist allow the user to display the same subset of entities of the nomenclature for each of the subjects, using a common global orientation. In our opinion, visualization of many subjects and structures, all at the same time, is essential to grasp the variability of the brain organization.

### Visualization Using *Anatomist*

Our lab has been developing the free and open-source 3D visualization software called *Anatomist* for about 25 years. It is able to display efficiently a large number of subjects and modalities simultaneously, either superimposed in the same view, or in separate views. It supports all kinds of neuroimaging objects: 3D/4+D volume images, meshes and textures, regions of interest, time series, fiber bundles, or structured compound objects such as cortical sulci, and it reads most common neuroimaging data formats. Objects may be displayed in different ways, and combined to display mixtures of objects.

#### A Few Words About *Anatomist* History and the Motivations to Develop It

**FIGURE 2:**
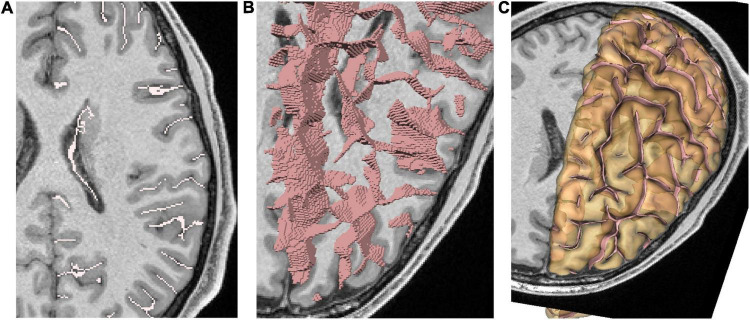
Representation of cortical folds using Morphologist; each fold is represented by a local negative cast of the cortex, usually corresponding to the cerebrospinal fluid, which fills up the fold. **(A)** 2D representation of folds. **(B)** 3D representation as voxels lists. **(C)** 3D representation as meshes. The hemisphere pial mesh is also displayed with slight transparency.

The ability to display, edit, and annotate structured data is one of *Anatomist*’s most original features, and constitutes what we call a ‘‘structural viewer.’’ These unique features have been developed specifically for the needs of our lab to work on structural anatomy. The original need was to build supervised learning databases for our cortical sulci identification algorithms (*Morphologist*,^1^ Riviere et al., 2002). Each cortical sulcus corresponds to a group of cortical elementary folds and is difficult to draw in a 3D T1-weighted MRI volume. Therefore, a dedicated preprocessing is used first to extract the folds one by one. Each such fold is represented by a local negative cast of the cortex, usually corresponding to the cerebrospinal fluid, which fills up the fold (Mangin et al., 2004a; see Figure 2). This representation is inferred from a 3D skeleton and corresponds to a set of voxels with a surface geometry (each voxel neighborhood splits the background into two different connected components). The representation usually extends from the brain hull to the deepest part of the fold. The number of folds automatically extracted by the *Morphologist* pipeline can vary according to the folding morphology but also because of segmentation instabilities induced by acquisition noise. A typical hemisphere leads to about 300 folds. All the extracted folds are embedded into a graph structure, which records not only the voxel-based representations but also the relative topography of the folds with each other (junctions and proximity relationship). A sulcus is made up of the aggregation of several of these folds, mainly because of sulcus interruptions and branches. Hence, all the folds belonging to a given sulcus are annotated with the same label.

In order to train an automatic machine learning system, we had to annotate the folds of a set of subjects using a sulcus-based nomenclature. Hence, we had to browse each graph in 3D in order to annotate the folds (the graph nodes) according to their localization, shape, and topography. This was one of *Anatomist*’s first use cases. The goal was to display the folds as colored meshes in 3D, load and display nomenclatures, and select and edit graph nodes (folds in this case) to perform the annotation: assign names from a nomenclature to graph nodes and of course, save the modified graphs.

To build a consistent database, we also needed to display and annotate sulci from several subjects side to side, which led to the inter-subject atlas navigation features.

*Anatomist* has been developed from the start with a wide genericity in mind, and the features developed for sulcal anatomy can be reused in a similar way for different structural studies: sub-cortical nuclei, axonal fiber bundles, or any other kind of structural representation. The next section discusses the suitability of *Anatomist* to deal with structural atlases.

## Structural Atlas Browsing Features

### Brief Presentation of *Anatomist* Features

As a general usage viewer, *Anatomist* is not directly set up for every use case, but it is developed both as a user-oriented application and as a software library, so it can be used to easily build case-specific custom applications, and extension modules can be added. *Anatomist* differs from other visualization software commonly used in the field (some are part of popular software packages, such as *Freesurfer*, *FSL*, or *SPM*) in several ways. First, it does not impose a hard-coded views layout (typically 3 or 4 views for the axial, sagittal, and coronal views) but allows the user to open as many customizable views as needed. Second, it is designed to work with “structural objects,” which may have a complex graph structure, and allows the user to edit and annotate such objects. Third, its coordinate transformation system does not impose a standard common coordinate system but allows free transformations between objects.

Such flexibility makes it appear a bit more complex to handle at first than more classical software, but concepts are quite simple, and it is rapidly taken in hand. Moreover, dedicated applications can easily be developed and hide some of the complexity: as an example, a classical simplified 4-views application variant is proposed with *Anatomist* (under the name “*AnaSimpleViewer*”).

*Anatomist* is free and open source, and its source code can be found on *GitHub* repositories^2,3^ (separated for licensing reasons).

It relies on several other open-source libraries; some also developed in our lab and available in *GitHub* repositories (the *AIMS* image manipulation library, for instance) and other general libraries (*Qt* for graphical interfaces, *OpenGL* for 3D rendering, *PyQt* for python bindings).

Binary distributions can be downloaded on: https://brainvisa.info.

In addition to loaded objects, it is possible to create new objects, which correspond to interactions between several existing ones. For instance, to display a 3D or 4D functional activation volume onto a gray/white matters interface mesh, it is possible to load both the mesh and the functional volume, and then create a mixture object from them. This mechanism is called “fusion” in *Anatomist* and offers a large panel of display possibilities; about 25 types of “fusion objects” are handled. *Anatomist* features are not limited to visualization; several edition modules allow the user to interact with the displayed data, like regions of interest drawing, painting on a mesh surface, or structural data edition, which will be discussed later in this paper.

#### Coordinate Systems

Every object and view in *Anatomist* is assigned a coordinate system (a referential), which can be shared with other objects or views. Affine transformations may join referentials in a transformation graph. Coordinates assigned to referentials, which are linked, directly or indirectly, *via* transformations, are transformed automatically on-the-fly (possibly combining several transformations), and taken into account in object interactions inside “fusion” objects.

This system does not assume a central coordinate system where each transformation would necessarily go to; data from multiple subjects or several modalities may have transformations from one modality to another for each subject, and then one subject modality may have a transformation to an inter-subject template or atlas, etc. This allows the user to load and manipulate each data in its native coordinate system but also to display it in any other referential. This way, we can get out of the classical paradigm where data are normalized and warped into a common space. Individual specificities are preserved.

### Multimodal Data Visualization

*Anatomist* views may display any data and combine them. Complex scenes can be built to display multimodal data, acquired from different devices, each endowed with its coordinate system.

**FIGURE 3:**
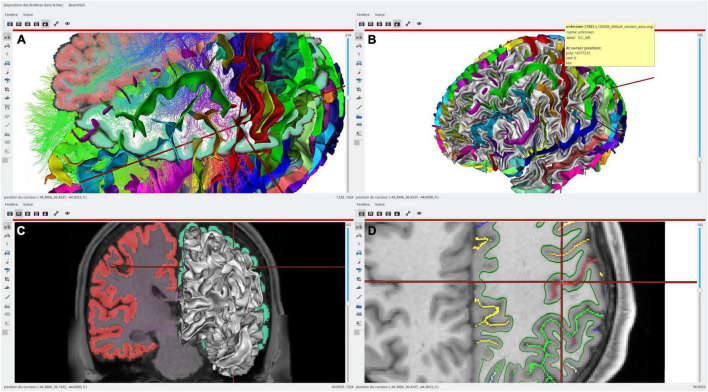
An example of single subject, multimodal data visualization using *Anatomist*. On this example, we have displayed: (**A**, top left) a skull-stripped T1 MRI slice and Freesurfer segmentation overlaid, a cortical sulci graph in 3D obtained using Morphologist, a subset of a tractography reconstructed by MRTrix, a clipped gray/white matters interface mesh with Desikan cortical regions obtained by Freesurfer; (**B**, top right) the brain gray/white interface mesh and cortical sulci; (**C**, bottom left) a skull-stripped T1 MRI slice and Freesurfer segmentation overlaid in coronal orientation and a left hemisphere gray/white mesh; (**D**, bottom right) a skull-stripped MRI and a 2D representation of cortical sulci in axial orientation, with the gray/white mesh intersection on this plane (green). The objects live in different coordinate systems for acquisition and/or processing reasons (different kinds of software are processing using different spaces), and affine transformations between them have been loaded in *Anatomist*. Object’s location and cursor position match in all views.

Figure 3 illustrates such a use case on a single subject, a multimodal situation where several kinds of processing software have been used; T1 MRI data have been processed using *FreeSurfer*^4^ for segmentation and brain meshing; *Morphologist* (see text footnote 1) has been used to extract and identify cortical sulci, and *MRTrix*^5^ for diffusion MRI processing and tractography. Processed data have been combined in different ways (here, a skull-stripped brain MRI, a segmentation volume, gray/white interface surface meshes, a Desikan parcellation texture, cortical sulci, axonal fibers tracts). Each of the modality data and each software are using their own coordinate system, and displayed data are each in potentially different coordinate systems, which are non-trivially linked *via* affine transformations.

### Structural Viewer and Editor

The structural edition capabilities were one of the first motivations to develop a structural 3D viewer. Structured object sets can be manipulated as graphs. Graphs contain nodes and optionally, edges linking nodes, each of these elements holding editable properties and 3D objects such as meshes and/or voxel sets. For instance, in sulci graphs, each node represents a cortical fold. They can be displayed in *Anatomist* and selected in 3D views. When a graph node is annotated from a hierarchical nomenclature, it can be displayed with a distinctive color, which refers to the entity annotating it. The graph and its nodes and edges can also be displayed in a “structural view,” which allows the user to browse the object sub-elements and properties. Elements and properties can be edited. To facilitate the node annotation, an interface allows the user to assign a label from the nomenclature in several ways without manually typing it to save time and avoid mistakes. One way consists in selecting the structure label in the nomenclature and sending it to the selected graph node property. Another more convenient way allows the user to perform a copy/paste operation; a label can be picked from a graph node, graphically in 3D views, from the same subject or another one, or from a 3D model visually displaying the full nomenclature, and pasted onto a series of selected nodes in the edited graph, using a single keyboard action.

**FIGURE 4:**
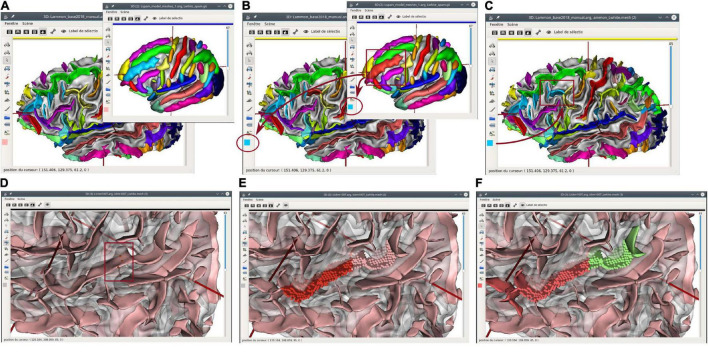
Structural edition capabilities in *Anatomist*. Top: copy/paste label operation. **(A)** A sulcal graph (left hemisphere) and a sulci model representation are displayed in *Anatomist*. **(B)** The middle frontal sulcus is selected on the model, and its label copied; its cyan blue color appears on the lateral bar of all views. **(C)** A fold is selected on the subject sulcal graph, and the label is pasted on it; its color switched from yellow (intermediate pre-central sulcus) to blue (middle frontal). Bottom: fold cut and split operation. **(D)** An unlabeled sulcal graph is displayed; we want to split a large fold belonging to both the superior temporal sulcus, and the anterior ascending terminal branch of this sulcus. In “fold split” mode, we draw a few points materialized by red bullets (inside the red rectangle); **(E)** the cut operation follows a line going through selected points and splits the fold graph node into two new ones. Mesh representation is replaced with a simpler “voxels list” representation until meshing is processed again; **(F)** the new folds are labeled using the copy/paste features.

More advanced edition features have been developed for structural regions such as cortical folds, like the ability to split a fold along a cutting line drawn semi-manually going through one or several selected points. This tool was needed to overcome segmentation imperfections, or anatomical specificities. It is generally used when two sulci are erroneously merged into a single fold node by the *Morphologist* segmentation pipeline. After the splitting operation, the graph structure is updated; new nodes and edges are added to the graph. Figure 4 illustrates such a use case.

The structural edition tools have allowed our team to build and maintain structural cortical sulci atlases, which are used to train supervised Machine Learning algorithms. The resulting sulcus recognition tools are integrated in *Morphologist* and have been applied to more than 50,000 brains. This application of *Anatomist* is still useful, as training databases continue to evolve and grow, in order to feed several generations of pattern recognition algorithms.

This structural edition tool is also useful outside of our team and the training database application; for studies where identification is crucial, users familiar with sulcal anatomy are using *Anatomist* to check and correct the automatic labeling before proceeding further with downstream operations. Ease of use and clarity of the interface is essential for this kind of application. Such an application can be, for instance, a morphological study, which statistically compares measurements on identified sulci between two population groups (healthy/pathological, or right handers/left handers, etc.). To do so, the accuracy of sulci identification is important. For large studies (about 50 subjects and more), manual corrections are generally not needed to reach statistical robustness, but for smaller studies, performing manual corrections on sulci labelings helps obtain cleaner, sharper, and more significant statistics. Several groups have performed studies in psychiatric and neurological pathologies studies; Molko et al. (2003), Plaze et al. (2009), and Mellerio et al. (2014) are a few examples where manual corrections have been helpful.

The sulcal analysis use case is one of the most used, and thus should be explained briefly; to build a sulcal graph, the *BrainVisa/Morphologist* toolbox can process a raw T1 MRI brain image, segment the brain tissues, and extract a cortical sulci graph (Mangin et al., 1995). The software then can apply an automatic sulci identification model, which provides labels for each sulcal graph node. The most recent algorithm (Borne et al., 2020) is also able to automatically split nodes, which should belong to two or more sulci. A set of subjects can be processed sequentially or in parallel using this toolbox. When sulcal graphs are obtained, they can be browsed, visualized, and edited manually using *Anatomist*, which can be triggered from the *BrainVisa/Morphologist* application using a single button click. It shall be noted too that if *Morphologist* is the only tool designed to build sulcal graphs from segmented brain images, it is not the only brain segmentation tool. Several kinds of software may be used for segmentation and preprocessing, and then segmented images may be imported in Morphologist to build sulcal graphs. This importation may require some adaptations in some cases, and links have been written with the *Freesurfer* software (see text footnote 4), for instance. A sulcal morphometry tool allows the user to get measurements on identified sulci, since sulcal graphs embed a series of preprocessed measurements, such as sulci sizes, lengths, depths, average cortical thickness, sulcal opening, etc. Population-level statistics can then easily be made on a per-sulcus basis.

Other structural representations may be used and manipulated exactly the same way in *Anatomist*, like subcortical nuclei, brain parcellations, or fiber tracts bundles. The graph representation is generic enough to hold, save, load, and display different kinds of data for different applications. The same principles work to display and edit such data, even if most of these other applications do not require the identification edition tools.

### Structural Atlas Navigation

Human cortical anatomy is complex and deeply variable. To grasp and study the full cortical variability, an average map is obviously not enough. For this purpose, we need to display and compare many subjects, side to side, in a common rough orientation but without non-linear deformations in order to keep intact anatomical shapes for every subject. This is where the structural atlases, as we have defined them, are needed. Dealing efficiently with such atlases requires the ability to display and navigate across many subjects. The *Anatomist* can load and display many subject data, and allows a common view orientation and synchronization (either punctually or constantly synchronized). *Anatomist* does not impose a limit, as long as the machine it runs on has enough memory and screen display capacity. On standard computers, it is possible to load, for instance, at least a hundred cortical sulci graphs. But display screens are actually a limit, because showing hundreds of brains on the same screen will display each one too small, and we may also reach 3D hardware memory and computing limits. Loading time for the data may also be a burden. In practice, display will significantly slow down when more than 20–30 brains are displayed on a standard computer (depending on the machine and GPU capabilities).

**FIGURE 5:**
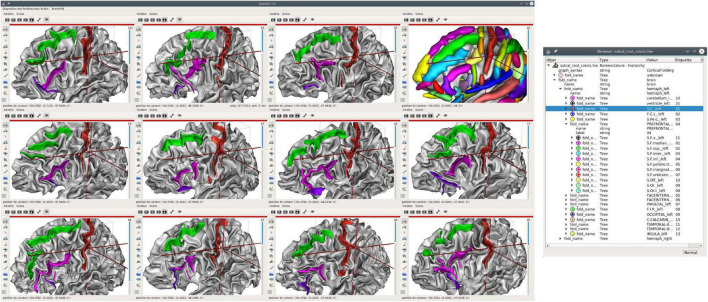
Structural atlas browsing, displaying the same entities on many subjects; here, 11 left hemispheres are displayed, each with 3 sulci: central sulcus (selected, in red), superior frontal (green), and inferior frontal. The latter is made up of two distinct entities, the anterior (purple) and posterior (pink) parts. The sulcal model is also displayed in the upper right 3D view. A structured display of the hierarchical nomenclature is shown on the right. All 3D views are displayed using the same orientation in a common space, although each piece of data lives in its own native coordinate system.

**FIGURE 6:**
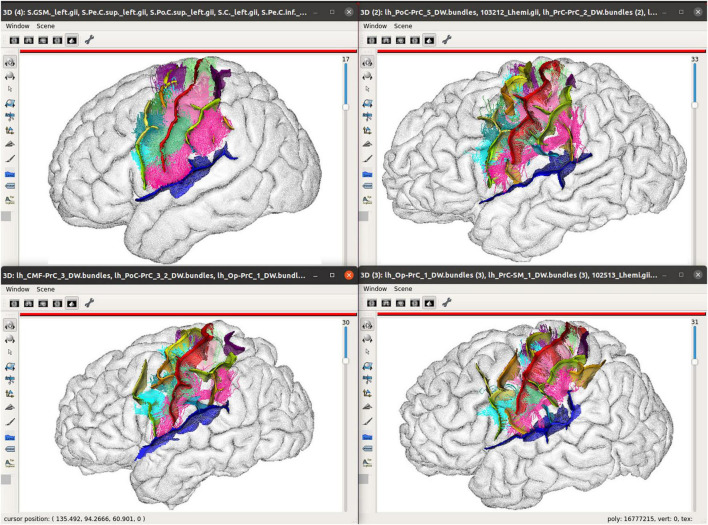
Multimodal structural atlas browsing; A subset of sulcal graphs and labeled fiber bundles are displayed simultaneously for three different subjects and an average model in a selected region of the brain. The top left view displays the sulci computed from the average icbm152 MRI using Morphologist and a few of the short bundles of an 80-subject atlas (Labra et al., 2019). The other views show instances of the sulcus and bundle structural atlases in 3 individual subjects, each in its individual native space. Sulcal and bundles data are displayed with nomenclatures, which are used to identify the same structures across subjects, and assign colors to them. Hemisphere brain meshes are displayed in a wireframe mode, here, to allow a better visualization of the fiber trajectories, which circumvent the folds.

To navigate structural atlases, it is thus alternatively possible to display brains by a subset, in a kind of a paginated view. Dedicated tools may be easily developed from *Anatomist*, which is usable as a programming and scripting library to build custom applications. The *Morphologist* toolbox of *BrainVisa* software suite provides this kind of display applications, and Figure 5 illustrates such a use case for cortical sulci comparison. Figure 6 is another illustration, which combines both the inter-subject atlas display and the multimodal aspect; it shows both sulci and labeled fibers bundles (Labra et al., 2019) in a specific region, for several subjects, and also for an average subject. The fiber bundle atlas has been established using sulcal-based non-linear (iconic) alignment of images, and a clustering algorithm for the fibers. Then, individual subject fibers have been labeled in relation with the atlas, and displayed in their native space. As a matter of fact, the algorithm is using a combination of structural and iconic approaches, and ends up with a structural result.

### Screen Walls

For large structural atlases, it is also possible to make use of screen walls, which offer a very large display space where hundreds of brains can be displayed at the same time. Using a screen wall generally requires adapted tools to efficiently operate computer and 3D hardware.

Some derivations of *Anatomist* scripts have been written for a wall of screens application. The *Maison de la Simulation* (*MDLS*, Paris Saclay, France) has developed a tiling web application “*TiledViz*” (Mancip et al., 2018), which allows us to display on its wall of screens a rather large number of views, each in a display ‘‘tile.’’ Tiles are rendered on the large screen wall (or alternately on smaller ones), and each one is a containerized application (using technologies such as *Docker*,^6^ or *Singularity*^7^), which can run on separate nodes of a small cluster, or can technically run on any remote machine, thus allowing parallelization. The system supports displaying several hundreds of tiles. We have adapted in a *TiledViz* case, named *TiledAnatomist*, such tiles to each run an *Anatomist* instance displaying one brain.^8^ The application has a network connection, and a master controller dispatches communications to all instances to propagate events from one container to the others, or to remotely pilot each instance. This allows the system to achieve view cameras and selection synchronization between tiles in real time. All Anatomist edition tools are, of course, available in this tiled mode.

When multiple computers are used to render tiles in a network, each machine computes renderings for a limited number of brains (tiles), and *TiledViz* combines tiles on a single large screen, a wall, or multiple screens, while enabling communication between the machines. Each has an overhead, mainly due to network traffic for the rendered images processed remotely by each machine, but the tiled application is able to scale to hundreds of brains, while maintaining a reasonable rendering speed.

**FIGURE 7:**
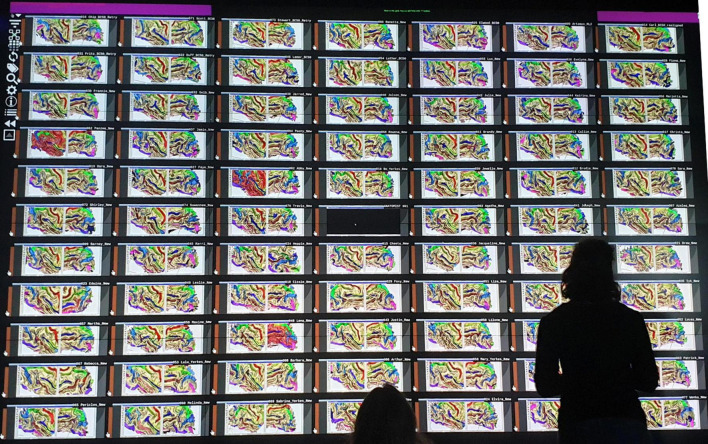
A fold labeling session in front of a wall-size screen to design a chimp-based structural atlas of cortical folding.

This tiled application used at the “*Maison de la Simulation*” allowed us to build and polish our last sulci atlas, used for training the latest generation of sulci identification models, including CNN approaches (Borne et al., 2020). Our databases sizes were 60–80 subjects, each having two separate views for left and right hemispheres. All the subjects could be seen simultaneously by a group of experts, which was very important to achieve a consistent consensus across the human expert annotations of sulci (see Figure 7). Several experts could discuss while looking at the entire dataset, which prevented drifts occurring with the paginated strategy. The wall-based approach overcame the weaknesses of the earlier training atlas (Perrot et al., 2011), which contained contradictory identifications that could not be detected when all the brains could not be displayed side to side. Indeed, the sulcal patterns are very complex, and require a good expertise to be identified; and even experts do hesitate or propose conflicting opinions. The latest generation is thus far more consistent and contributed significantly to build a more accurate pattern recognition model and atlas (Borne et al., 2020).

### Technical Aspects

#### Library and Languages

*Anatomist* has been developed as a C++ library, with a user application. On top of this C++ library, Python language bindings have been added, and extensions are developed in either language. Of course, Python, as an interpreted language, is more flexible, does not require to deal with compiler and system compatibility issues, and allows simpler and faster development. To summarize these technical aspects, the C++ core ensures an efficient and fast rendering engine, based on *OpenGL* with hardware 3D acceleration, and Python allows very easy handling and extensibility of the library.

#### Cross-Platform Source Code and Container Distribution

*Anatomist* and its underlying libraries have been developed as cross-platform code; the source code can compile and run on Linux, Windows, or MacOS systems. However, since version 5.0, the binary distributions are Linux-based containers (*Singularity* images, or virtual machines built for *VirtualBox*). This virtualization has several advantages and a few drawbacks. It primarily reduced the amount of maintenance and simplified the packaging and installation tasks but also provides a binary compatible distribution for all systems and developers, and achieves better reproducibility of the software on heterogeneous systems. Toolbox developers do not have to bother about portability any longer and do not need to compile their code on many architectures to distribute them. Drawbacks include slight performance overheads (quite limited), and possible issues using 3D hardware for *Anatomist* rendering. Containers and virtual machines allow the user to use 3D hardware in some hardware/system combinations, but not in every case. In such a situation, software rendering is the last option, but it works well and reasonably fast on today’s machines.

#### Structural Browser Handling and Libraries

The use cases described in this paper, mainly showing cortical sulci applications, can be used in other contexts. The applications described are generic enough and rely on two data structure concepts: graphs and nomenclatures. A graph is a set of nodes, optionally linked by edges, as described in section “Structural Viewer and Editor.” The structure definition is general enough to represent any object sets with or without relations, and *Anatomist* is able to display graphical objects (meshes, voxels lists, etc.) stored in nodes. Thus, it can be used to represent cortical folds, fibers bundles, sub-cortical structures, functional activation regions, or any regions of interest sets. File formats have been designed for such objects to store and exchange them, and libraries allow users to build, modify, or manipulate them. Nomenclatures are simpler objects; they are hierarchical tree structures where each element represents a brain structure, with a name, and, possibly, a color to be assigned to objects with this name. Nomenclatures also have file formats and manipulation libraries. A simple interaction between graphs and nomenclatures allows us to display colored objects sets and to navigate between them across subjects in *Anatomist*. For the programmer, a C++ library is available, and Python bindings allow us to manipulate graphs and nomenclatures just like python dictionaries objects, with additional convenience functions to ease storing graphical objects in graphs, insert or delete nodes or edges, etc. Once saved, a new kind of graph will be used in Anatomist the same way sulci graphs are used.

## Use Cases and Discussion

### Structural Atlas Is of Interest for Various Needs

What we call structural atlas is not a rare need, specific to our research program. A growing number of research groups and studies are taking interest in fine individual brain architecture and its variability (Wang et al., 2015; Glasser et al., 2016; Gordon et al., 2017a,b; Bijsterbosch et al., 2018; Kong et al., 2019; Seitzman et al., 2019). As studies get more precise and subtle, individual structural comparison attracts more researchers than it did a decade earlier. Deciphering the variability of the cortical folding pattern is the main topic of interest in our group, which is thus highlighted in this study, but other applications can benefit from browsing inter-individual structural atlases with many subjects. We can cite, for instance, studies aiming at understanding and classifying axonal fiber bundles, subdivisions of deep nuclei, different kinds of brain or cortex parcellations, or individual functional regions. Comparing microstructural parcellations (cytoarchitectonic areas) may also fall into this type of study, even if the number of subjects in such studies is not as high as for *in vivo* MRI studies.

Another aspect, which has been somewhat left aside for many years, partly for lack of exploration tools, is interindividual multimodal comparison of the above structural aspects. Exploring precise relations between connectivity, sulco-gyral folding (like in Figure 6), function, genetics, and maybe microstructure is a challenge that needs powerful, versatile, and adaptable visualization tools like *Anatomist*. The classical iconic approach, even using fine statistical maps, will not provide sufficient information to decipher inter-individual relations between structures seen through different modalities.

A number of large-scale studies have successfully used sulci analysis. Some have crossed sulci analysis with genetics or heritability data (Pizzagalli et al., 2020, 9,000 subjects processed; Le Guen et al., 2018, 820 subjects processed; Le Guen et al., 2019, 15,600 subjects processed; Le Guen et al., 2020, 20,000 subjects processed; Karkar et al., 2021, 16,300 subjects processed). The CATI neuroimaging services^9^ have processed over 20,000 subjects mainly for multicentric studies focused on aging and neurodegenerative diseases.

Several of the above studies have used at least partly the same subjects and have reused the same processing results, especially those using the UK Biobank^10^ and HCP^11^ cohorts, but in total, as stated in section “Structural Viewer and Editor,” more than 50,000 individual brains have been processed using the sulci segmentation, identification, and analysis tools.

### Inter-Species and Brain Development Comparisons

**FIGURE 8:**
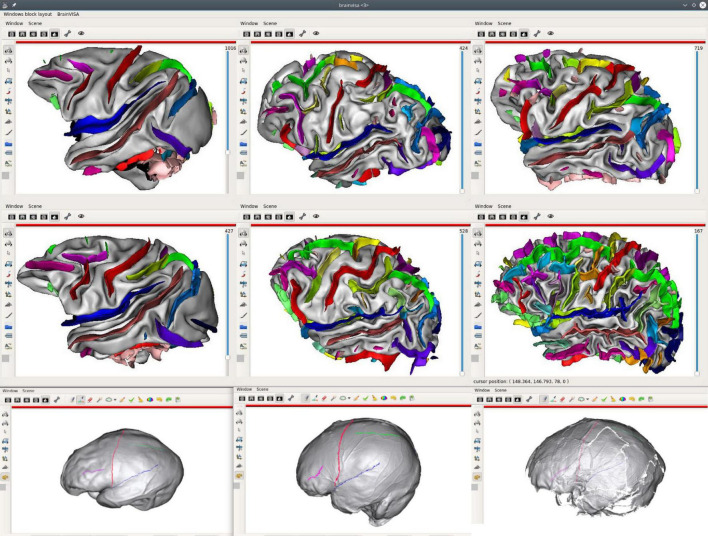
Inter-species sulci comparison structural atlas; the first two lines of the figure display sulci of 6 different species (from left to right, and then, top to bottom: a macaque, a pongo, a chimpanzee, a baboon, a gorilla, a human). Sulci have been manually identified on these brains, using a common sulci nomenclature, in order to get structural correspondence between subjects/species. The last line extends the comparative study to extinct species using skull endocasts, using another Anatomist feature, allowing to draw sulcal lines (Le Troter et al., 2012) [from left to right: Sinanthrope XII (an Homo erectus), a recent Homo sapiens, La Ferrassie 1 (an Homo neanderthalensis] (Balzeau and Mangin, 2021).

Structural atlases are also useful to investigate inter-species brain similarities. Some homologous cortical structures (sulci and gyri) have been established between, for instance, humans and several ape and monkey species, but some of them do not match. Warping and dense 3D point-to-point correspondence do not work (or not well) for this kind of studies, and we need to display matching (or potentially matching) structures with native shapes and details, in a roughly common orientation, in order to hypothesize whether structures are the same in different species. Figure 8 illustrates such a use case for the comparison of the sulci of several monkey, ape, and extinct species.

In addition to species comparisons, studying the human brain growth and development from early gestational age to adult age is also a key topic with a dynamic research community. Acquiring brain MRI images from infants, preterm, and even prenatal subjects is now possible. Some of the segmentation preprocessings may differ from adult MRI processing, but obtaining sulcal graphs for infant brains is possible, and allows the user to obtain the same representation as for adults. Some structural infant atlases have been built and have been used to train a specialized recognition model in order to improve automatic sulci identification. Some studies are using these atlases and tools to help understand the brain folding process and to try to explain the variability observed at adult age (De Vareilles et al., 2021).

### Iconic and Mixed Structural Approaches

The iconic methods are, of course, not impossible to use in *Anatomist*, and combined visualization strategies can be useful; one can display individual structural data alongside iconic atlases or templates. Affine coordinate transformations are taken into account; thus, any affine registration or normalization information can be handled natively in *Anatomist*.

**FIGURE 9:**
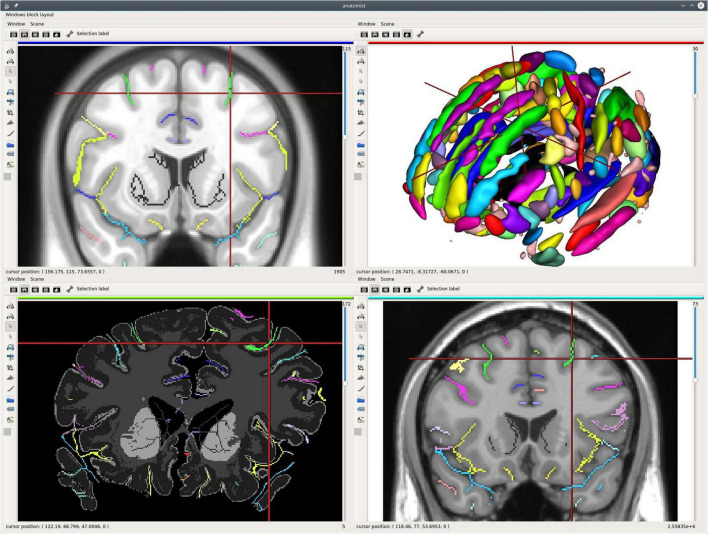
Non-linear correspondence between different views and data. Three atlases and a sulci model representation are displayed. The ICBM152c asymmetric (top left), BigBrain (bottom left), and Colin27 (bottom right) templates are displayed, each in its native space, each with its own colored sulci set extracted using the Morphologist pipeline (in 2D here). Non-linear coordinates transformations apply between cursor positions in each view, thanks to DISCO/DARTEL transformation fields. The cursor position set by clicking on the superior frontal sulcus on the Colin27 image (bottom right) thus points to the same sulcus in the other templates’ views in different spaces, even when the atlas has large deformations such as on the postmortem BigBrain. Additionally, the sulcal model of Morphologist (top right) aligned to the ICBM152c space also displays the same sulcus at the cursor position.

Non-linear transformations are not handled natively in *Anatomist* for three reasons: (1) they cannot be integrated in an *OpenGL* pipeline, (2) such transformations may not be totally inversible at every point, which may cause issues in the rendering pipelines, and (3) they are costly and would notably slow down the whole rendering engine. Thus, such transformations are not applied on-the-fly in *Anatomist*. However, they can be handled in a custom application; *Anatomist* event system can react to mouse clicks or other user interactions. The custom application can non-linearly transform the click position coordinates, and then adapt other displays to visualize the corresponding location in other atlases and subjects, possibly aligning them in the “closest” affine orientation, without non-linearly resampling all objects. This preserves data specificity (no warping) but keeps, however, a precise local correspondence, without the cost of data resampling. This use case has been implemented in a little side application named “*ana_atlas_nonlin.py*” (Figure 9) and shipped with the version 5.0.1 (and later) of *BrainVisa/Anatomist*.

The script can load non-linear transformation graphs and use them to work as the non-linear point-to-point correspondence. It is also able to select regions (objects) by name, select the corresponding structure in every view, and focus the view on it, should it be in an individual subject or in an atlas. It, therefore, works as a hybrid iconic/structural viewer. The user may use it with the deformation fields between the MNI ICBM152 2009c template atlas, the MNI Colin27, the BigBrain atlas, and an infant template atlas. Those may be found on the *Human Brain Project* (HBP) knowledge graph at the following URL: https://search.kg.ebrains.eu/instances/Dataset/7a9aa738-a5b2-4601-818e-05db2627ba5c. The actual atlases can be downloaded by following the links on the HBP URL above. The deformation fields between the atlases have been obtained using the DISCO/DARTEL registration methods, with sulcal alignment constraints (Lebenberg et al., 2018), which promotes the correspondence between the main sulci during alignment.

This application is mainly a toy; it has been developed within a few hours to show the possibilities of *Anatomist* as an atlases and subjects navigation tool, and also to exhibit its scripting and extension capabilities. However, it is already useful in its current state and can, of course, be completed to end up with a more complete, “serious” application. Figure 9 illustrates this application.

### Comparisons of *Anatomist* With Existing Software and Libraries

*Anatomist* is not the only visualization software available in the field of neuroimaging. Several software packages, such as *SPM*, *FSL*, or *Freesurfer*, provide their user-oriented viewer application. Most of them are good display solutions for classical, iconic visualization, but in our opinion, none of them offers the full features required for structural atlas browsing and edition. Namely, apart for displaying 3D or 4D volumes, and surface meshes, the features we expect are (1) the ability to display as many subjects as the user wants (or the display screen limitations impose), (2) structure selection and a link between subjects from a nomenclature, (3) using complex transformations graphs between several coordinate systems, (4) structural edition, (5) scripting and programming possibilities to design custom focused applications.

*SPM*^12^ is one of the most popular brain image analysis software (and one of the first historically available), developed in Matlab language. Its visualization module is limited to classical 3 orthogonal views, 2D rendering of volume, and it assumes all coordinates’ transformations going to the same central space.

*FSL*^13^ is also a widely spread analysis software and features *FSLeyes*, which is a 3D viewer enabling the display of 3D/4D images and meshes, and has some flexibility on views and layout, but is more oriented to pre-selectable layouts rather than to full customization. Moreover, it also assumes a central common coordinate system to get from an image space to another. It has a Python language application programming interface (API) for customization.

*Freesurfer* (see text footnote 4) is another very popular neuroimaging analysis software and proposes *FreeView* and *TkSurfer* for visualization. As software oriented toward surface-based analysis, its visualization is 3D. The interface proposes a few preset views’ layouts, which are easy to use but limited in customization. *FreeView* is not designed for scripting and extension programming, and *TkSurfer* can be scripted in Tcl language.

Other software libraries and toolkits provide programming tools and functions to develop visualization: *VTK* and *Mayavi* can be cited. *VTK*^14^ is a C++ language library dedicated to 3D visualization. The *VTK* project started approximately at the same time our group started the *Anatomist* project, and there are similarities between them. *VTK* has a well-designed API and offers powerful 3D rendering features and interactions. The C++ library has Python language bindings. It does not provide a user application; thus, it is intended for programmers, not directly for users. *VTK* is a general visualization library, and is not primarily designed for neuroimaging; however, it has neuroimaging applications, and it supports a few neuroimaging image formats. But getting started with *VTK*, especially for neuroimaging applications, is more difficult than with *Anatomist*. *Anatomist* used to have a *VTK* plugin, which allowed to mix *VTK* and *Anatomist* objects in a “hybrid” rendering engine. As users were not using this plugin, it fell into obsolescence.

*Mayavi*^15^ is a scientific data visualization library in Python language. It proposes a user application and is not dedicated to neuroimaging. In fact, its real added value is in displaying many kinds of plots or time series in a nice Python API, where, for 3D rendering, it relies on *VTK*.

*3D Slicer*^16^ is 3D visualization software developed in C++ language, and is dedicated to medical imaging (although not precisely for brain imaging). Its focus is on image processing; it features segmentation or registration algorithms, but also provides powerful 3D rendering features. It is also based on the *VTK* library. It provides a C++ API for extensions, but (in our knowledge) no scripting language API; thus, customization is more laborious than in Python language.

The *Human Brain Project*^17^ develops a web-based viewer,^18^ which features displaying several species (humans, rats, mouses), each containing multiple atlases (for humans, the MNI ICBM152 template, the MNI Colin27 template, and the BigBrain multiscale atlas) with sets of regions (cortical layers, cortical areas, fiber bundles). The viewer includes a very efficient multiscale view engine, which supports zooming fluently into very large microscopic datasets (used for the BigBrain histological data). Coordinate transformations are, for the moment, handled using pre-resampling of the atlases. The developers plan to support uploading of user data to display overlays of the various atlases on user data. It is a fixed 4-view layout. This viewer is convenient to explore the different atlases and regions sets. However, it does not include a multi-subjects comparison possibility, and even atlases cannot be compared side to side in this layout; the navigation application is not the focus of the HBP viewer.

To our knowledge, none of the cited software, and no other software in our knowledge, offers some kind of structural visualization and edition features like the ones present in *Anatomist*, and which actually qualifies for structural atlases browsing and manipulation.

## Conclusion

The use of brain atlases is a classical component of neuroimaging studies. Mapping data on existing atlases is a way of comparing individual data to the average situation. Visualizing multiple individual subjects for which some brain elementary structures have been extracted, to navigate across them for comparison purposes, is an important need when investigating structure variability. Structural atlases, and appropriate powerful visualization tools, are thus becoming mandatory to understand finer brain organization. Such atlases are also needed to train a wide range of machine learning algorithms; deep learning and multi-template patch-based algorithms, for instance, make use of a large quantity of annotated data.

Applications are found in several domains. Cortical anatomy, where structures are widely variable, and experts are even not sure of the structures to be studied, is the first application domain our team has been involved in. The applications extend from healthy adult brain cortex studies to related questions, like cortical folding mechanisms and sulci shape formation during brain growth, or comparison with other species, like chimpanzees, pongos, gorillas, baboons, and macaques, for instance. Inter-species single-map atlases are not possible to build, so comparing many subjects is necessary to establish links between species structures. Studying pathological brains folding is another related topic, where standard atlasing is not always the best approach; for instance, in epilepsy studies, even if patients may be roughly classified in categories according to symptoms and the expected location of epileptogenic zones, each case is unique and specific, and working with every individual subject, compared to others, is needed.

Then, other applications can also benefit from the same kind of approach. Axonal fibers, studied from diffusion MRI imaging and tractography, lead to fiber tracts reconstructions, which are difficult to interpret and validate, and the related macroscopic human connectivity is still unclear to date, especially for smaller and shorter bundles. Clustering and classifying fiber bundles, locating them in regard to connected regions, and comparing them between subjects, is one of today’s challenges. We can be also interested in comparing brain parcellations or other processing results, across multiple subjects. Multimodal comparison of different structures across many subjects is thus a concomitant question, where we need to discover how precise relationships between different kinds of structures vary or reproduce across subjects and across species.

Visualization is generally not considered the heart of methodological development, which is supposed to be theoretically driven by the adequation of mathematical models or algorithms to describe anatomical constraints, and then validated in regard to objective, “blind,” and impartial criterions (such as distances, DICE indices, etc.) and not by visual appreciation. However, in practice, looking at the data helps the researchers to establish the best processing algorithms and the best validation criteria.

Building the structural atlases evoked above involves, in many cases, the manual edition and annotation of the studied structures on many subjects. Thus, visualization and edition tools are required to build efficient processing algorithms.

Surprisingly, few software solutions are available to address this use case, which has no obvious and easy technical answer. Our visualization software library and application, *Anatomist*, qualifies, in our opinion, as a convenient and flexible solution to address this question, with many possibilities of extensions and customizations to build use case-oriented applications. Its efficient and scalable design allows the user to display large amounts of data, both individual data or atlases, with several navigation options (region based, coordinate position transformations, etc.). This possibility can help the neuroimaging community to go beyond the classical “normalization and averaging” paradigm and to grasp the complex variability of human and non-human brain structures.

## Data Availability Statement

The programs codes can be found here: GitHub (https://github.com/brainvisa/anatomist-free, https://github.com/brainvisa/ana tomist-gpl, and https://github.com/mmancip/TiledAnatomist). The standard templates deformation graph is available on https://search.kg.ebrains.eu/instances/Dataset/7a9aa738-a5b2-4601-818e-05db2627ba5c.

## Ethics Statement

Ethical review and approval was not required for the study on human participants in accordance with the local legislation and institutional requirements. Written informed consent for participation was not required for this study in accordance with the national legislation and the institutional requirements. Ethical review and approval was not required for the animal study because we just display, as an example, a few images of animals which were acquired for different studies.

## Author Contributions

DR led the development of *Anatomist*. DR and J-FM drafted the manuscript. NL and NV contributed to the fiber bundle use case. NL and AB contributed to the endocast use case. OF, BC, KL, OC, and WH contributed to the ape use case. YL and JL contributed to the non-linear use case. YC contributed to many software libraries in and underneath *Anatomist*. All authors have iterated on the manuscript.

## Conflict of Interest

The authors declare that the research was conducted in the absence of any commercial or financial relationships that could be construed as a potential conflict of interest.

## Publisher’s Note

All claims expressed in this article are solely those of the authors and do not necessarily represent those of their affiliated organizations, or those of the publisher, the editors and the reviewers. Any product that may be evaluated in this article, or claim that may be made by its manufacturer, is not guaranteed or endorsed by the publisher.

## Footnotes

1
https://brainvisa.info

2
https://github.com/brainvisa/anatomist-free

3
https://github.com/brainvisa/anatomist-gpl

4
https://surfer.nmr.mgh.harvard.edu/

5
https://www.mrtrix.org/

6
https://www.docker.com

7
https://sylabs.io

8
https://github.com/mmancip/TiledAnatomist

9
https://cati-neuroimaging.com/

10
https://www.ukbiobank.ac.uk/

11
http://www.humanconnectomeproject.org/

12
https://www.fil.ion.ucl.ac.uk/spm/

13
https://fsl.fmrib.ox.ac.uk/

14
https://vtk.org/

15
https://docs.enthought.com/mayavi/mayavi/

16
https://www.slicer.org/

17
https://www.humanbrainproject.eu/en/

18
https://atlases.ebrains.eu/viewer/
